# Attitudes to functional neurology and some other ‘prescriptive’ chiropractic techniques and their associations with chiropractic conservatism: a cross-sectional survey of chiropractic students

**DOI:** 10.1186/s12998-020-00308-7

**Published:** 2020-05-19

**Authors:** Marine Demortier, Guillaume Goncalves, Charlotte Leboeuf-Yde, Christine Le Scanff, Niels Wedderkopp

**Affiliations:** 1 CIAMS, University of Paris-Saclay, F- 91405 Orsay Cedex, France; 2 CIAMS, University of Orléans, F- 45067 Orléans, France; 3 Institut Franco Européen de Chiropraxie, 24 boulevard Paul Vaillant Couturier, 94200 Ivry sur Seine, France; 4 Institute for Regional Health Research, University of Southern Denmark, DK-5000 Odense, Denmark; 5 The orthopedic department, Hospital of Southwestern Jutland, DK-6700 Esbjerg, Denmark

**Keywords:** Neurologie Fonctionnelle, Techniques chiropratiques, Conservatisme, Ėtudiants en chiropraxie, Enquête, Functional neurology, Chiropractic techniques, Conservatism, Chiropractic students, Survey

## Abstract

**Background:**

Chiropractic technique systems (‘prescriptive’ techniques) might be interpreted as helpful guidelines. However, ‘prescriptive’ techniques, such as Functional Neurology (FN), Sacro-Occipital Technique, and Applied Kinesiology are more concerned with the ‘technical’ diagnosis than the condition and its symptoms and, thus, seem to provide easy solutions.

**Design and objectives:**

In a voluntary anonymous questionnaire survey carried out late 2017, we explored interest in ‘prescriptive’ techniques, particularly FN, among French chiropractic students in grades 3–6, and the possible link with chiropractic conservatism. We investigated their: i) attitudes to the use of ‘prescriptive’ techniques, ii) awareness of FN, and iii) attitudes to FN. Further, if their attitudes to some conservative chiropractic concepts influenced their clinical approach on iv) some ‘prescriptive’ chiropractic techniques and v) attitudes to FN.

**Method:**

Data reported in tables illustrated the prevalence of the use of ‘prescriptive’ techniques, awareness of FN, and positive attitude to FN (i.e. *interest in* and *acceptance of*). Students were given a FN score based on five questions on their *interest in* and *acceptance of* FN (0 to 5), dichotomized into two groups: ‘not positive attitude’ (0 to 1) and ‘positive attitude’ (2 to 5). Chiropractic conservatism was graded from 1 to 4. Associations were tested between conservatism groups and i) interest in ‘prescriptive’ techniques and ii) FN attitudes groups.

**Results:**

The response rate was 67% (*N* = 359), of which 90% were positive toward ‘prescriptive’ techniques. Only 10% had never heard about FN and in the 6th year all had heard about it. Only a minority, unrelated to the year of study, approved of the two examples given of FN concepts. Nevertheless, a majority were positive towards FN, more so in the higher grades. Students with the most conservative beliefs were 17 times more likely to want to use ‘prescriptive’ techniques and 11 times more likely to have a higher FN score.

**Conclusion:**

Although not taught in the curriculum, these students were attracted to ‘prescriptive’ techniques including FN, particularly in the higher grades. Curiously, despite this interest they do not generally agree with some key concepts within FN.

## RÉSUMÉ

**Introduction:**

Les recommandations de bonnes pratiques aident les thérapeutes à actualiser leurs connaissances et à exercer une pratique fondée sur les faits. En chiropraxie, les techniques chiropratiques dites systématiques sont décrites ici comme ‘prescriptives’. La Neurologie Fonctionnelle (NF), la Technique Sacro-Occipitale et la Kinésiologie Appliquée en sont des exemples, elles se concentrent sur un diagnostic ‘technique’ et ne prennent pas ou peu en compte la symptomatologie du patient. Ces techniques prescriptives – parfois utilisées dans des techniques chiropratiques se basant sur des concepts chiropratiques anciens – peuvent donc apporter aux thérapeutes des solutions facilement applicables en cabinet.

**Schéma et objectifs:**

À travers un questionnaire anonyme et volontaire réalisé fin 2017, nous avons étudié l’intérêt des étudiants en chiropraxie à l’Institut Franco-Européen de Chiropraxie (3-6^e^ années) pour les techniques chiropratiques ‘prescriptives’, plus particulièrement pour la NF; ainsi que sa possible association avec une vision conservatrice de la chiropraxie (mesurée par un score de conservatisme chiropratique). Précisément, nos objectifs étaient d’enquêter sur i) l’intention des étudiants quant à l’utilisation de techniques ‘prescriptives’ une fois diplômés, ii) leur connaissance de la NF et iii) leur attitude envers la NF (mesurée par un score de NF). Nous souhaitions également étudier si leur niveau de conservatisme était associé à iv) leur intention quant à l’utilisation future de techniques ‘prescriptives’ et v) leur attitude envers la NF.

**Méthode:**

La prévalence de l’utilisation des techniques ‘prescriptives’, de la connaissance en NF et de l’attitude favorable (i.e. *intérêt* et *acceptation*) pour la NF ont été reportées dans des tableaux. Un score de NF (0–5), calculé à l’aide de cinq questions sur l*’intérêt* et l’*acceptation* de la NF, a été attribué à chaque étudiant. Il a ensuite été divisé en deux groupes ‘attitude non favorable’ (0–1) et ‘attitude favorable’ (2–5) à la NF. Concernant le conservatisme chiropratique, le système de score est identique à celui utilisé dans le précédent report, nous avons donc obtenu quatre groupes, le quatrième étant le plus conservateur. Nous avons ensuite testé les associations entre les groupes de conservatisme et i) l’intérêt pour les techniques ‘prescriptives’ et ii) les groupes d’attitudes envers la NF.

**Résultats:**

Le taux de réponse était de 67%; l’échantillon est considéré comme représentatifs de la population étudiante. 90% des étudiants souhaitent utiliser des techniques ‘prescriptives’. Seulement 10% n’ont jamais entendu parler de la NF mais, en 6^ème^ année, tous en ont déjà entendu parler. Une majorité d’étudiants est favorable à la NF, d’autant plus au cours des dernières années d’études. Cependant, indépendamment de l’année d’étude, seule une minorité d’étudiants approuve les quelques exemples relatifs aux concepts de NF. Les étudiants les plus conservateurs sont 17 fois plus susceptibles de vouloir utiliser des techniques prescriptives et 11 fois plus d’avoir un score de NF élevé.

**Conclusion:**

Cette cohorte d’étudiants semble s’intéresser aux techniques ‘prescriptives’ ainsi qu’à la NF – non enseignées au sein du curriculum – d’autant plus pendant leurs dernières années d’études. Étonnement, malgré cet intérêt, ils n’adhèrent généralement pas aux quelques concepts clé de NF présentés.

## Introduction

Guidelines in medicine and chiropractic are usually developed by groups of clinicians with research experience, reviewed by experts and by other clinicians with relevant background [1]. They are useful to guide the clinician towards an up-to-date and evidence-based approach to diagnosis and treatment. The ideal result of the use of guidelines is that they can streamline diagnostic and therapeutic strategies to ensure that patients with similar conditions will be treated reasonably and similarly by clinicians at various points in the health care system. However, they are not meant to dictate strict prescriptive methods of approach without room for reflection and variation.

Some chiropractors use specific technique systems to guide the treatment. There are many such techniques [2]. These typically consist of a predefined analytic approach, which leads to a ‘technical diagnosis’, i.e. indicates where, how, and perhaps even in what order treatment should be done. The treatment will then follow in a predetermined manner, based on this ‘technical diagnosis’. In our experience, some chiropractors apply such technique systems to all types of patients regardless symptoms, letting the ‘technical diagnosis’ guide the treatment. In other cases, they are considered a help to deal with an overruling specific diagnosis (e.g. of discal hernia, spinal stenosis). This type of approach can therefore be likened to a ‘guideline’.

Examples of this are various techniques such as Gonstead X-ray analysis, which purports to show the clinician where and in which direction to adjust the spine, based on the identification of curves and angles of vertebrae on radiographs [3]. Another example of a visual analysis is that relating to a ‘short leg’ and the consequent indication on where in the pelvis treatment should be provided (e.g. the Derifield test) [4]. Hence, the symptoms are not necessarily important at this stage of the prescribed treatment.

Other techniques mix various observations, such as the Sacro-Occipital Technique, which combines a set of visual observations of body sway with other subtle observational findings to classify patients into three categories, which will guide the clinician into a set treatment program [5], once again not necessarily taking into account the symptomatic picture.

Applied Kinesiology is even more complex and is claimed to be useful for many things, such as to identify the area and direction to adjust [6, 7]. This is done by using muscle testing, comparing strong (i.e. properly ‘locking’ muscles) to ‘weak’ muscles, either by having the patient touch various areas of the spine, or by ‘challenging’ the vertebra to see in which direction it should be adjusted. This type of test is also used to indicate if the treatment was technically successful regardless of the symptomatic development. Thus, such techniques use various approaches, including simple observation to more complex tests, using several ‘objective’ parameters (angle, position, strength, length etc).

There are other, even more complicated treatment methods, such as Functional Neurology (FN). This more recent system utilizes a number of neurological tests used also in classical medicine but with a more subtle interpretation [8]. Chiropractors who use FN extend their scope of treatment to the nervous system including the brain, targeting, for example, dysfunctional groups of neurons that, purportedly, can be stimulated with various therapeutic approaches, including spinal manipulation [8]. Examples of indications of treatment with FN are neuro-musculoskeletal disorders, symptoms related to traumatic brain injuries, neurologic diseases or disorders, psychiatric disorders, and various neurologic or non-neurologic isolated symptoms. The treatments do not seem to be specific to the conditions but they are related to the examination findings, in such a way that patients with completely different conditions can be ‘prescribed’ very similar treatments [8]. A question we ask ourselves is: what delineates the chiropractors who believe in this extraordinary [8] and seemingly unproven concept [9]?

Although these techniques are all clearly ‘prescriptive’ in nature, the objective origin of such chiropractic ‘guidelines’ is unclear and seems to be based only on clinical observations and opinions without evidence for their validity. This does not mean that they have not been developed without reflection. A detailed description of the early development of some such ‘prescriptive’ techniques has been published elsewhere [2]. There has, however to our knowledge, never been a documented and published description of their development on a scientific basis and they seem to have been established without the support of a scientific reference group or after critical peer review. Nevertheless, in our experience, these kinds of ‘prescriptive’ techniques are very popular among some chiropractors and chiropractic students, presumably because most of them are easily performed and can be used on virtually all patients.

On reflection, we find the concept of ‘prescriptive’ techniques in chiropractic surprising for two main reasons. First, why would spinal problems be different from other human pathologies, i.e. how could they be unrelated to symptoms and underlying pathologies? Secondly, the human body is complex and unlikely to fit in with some ‘prescriptive’ technique, which seems self-evident. Why then, are some chiropractors so interested in this ‘simplistic’ approach?

It may be difficult to study questions like this in chiropractic populations, because some chiropractors might find the topic offensive [10]. However, it was thought to be less problematic to study this among chiropractic students, who may not feel the need to ‘protect’ the reputation of their future profession. Thus, in a recent survey from Australia, 82% of chiropractic students were interested in using ‘prescriptive’ techniques [11].

An additional peculiarity for the chiropractic profession is that there are clinicians, who follow the long-standing chiropractic tradition which states that spinal disturbances (‘subluxations’) may interfere with the inner life force of the body (‘Innate Intelligence’) from expressing itself fully, which in turn may cause disease and that the removal of such disturbances through spinal manipulation can heal a multitude of disorders [12]. According to a study of chiropractic students in Australia, more than half thought that chiropractic spinal adjustments can “make it easier to give birth” and that they “help the immune system” [13]. In a recent report on chiropractic students in France, such ‘conservative’ beliefs that align with the traditional Palmer Postulates were very strongly associated with an inability to identify non-indications to chiropractic treatment [14]. We thought it reasonable that chiropractors who have such a conservative approach to health and health care may likely be interested also in ‘prescriptive’ techniques and unusual treatment methods.

Again, it seems appropriate to study this topic in students, as they are the future generation of chiropractors, and although many receive their education in an evidence-based institution, it is evident that some are interested in various ‘prescriptive’ techniques, including the more complex varieties, such as FN. We therefore performed a survey to learn more about chiropractic students’ thoughts on ‘prescriptive’ chiropractic techniques including FN. The specific objectives were to investigate i) chiropractic students’ attitudes to the use of ‘prescriptive’ techniques, ii) their awareness of FN, and iii) their attitudes to FN. Further we wanted to determine if their attitudes to some conservative chiropractic concepts may influence their clinical approach in relation to iv) ‘prescriptive’ chiropractic techniques, and v) FN.

Additional information from this same survey has been reported elsewhere [14]. This was in relation to students’ clinical approach to contra-indications, non-indications and indications to chiropractic treatment and how this is influenced by chiropractic conservatism views.

## Method

### Setting and ethics

This voluntary and anonymous survey was conducted on French chiropractic students attending the 3rd to the 6th year of study at the *Institut Franco-Européen de Chiropraxie* on both its sites in Paris and in Toulouse. This school is, from 2016, a full time five-year course (previously full time six years), which is accredited by ‘Le Ministère des Affaires Sociales et de la Santé’ [15] and by the European Council on Chiropractic Education [16] and the school does not encourage the use of ‘prescriptive’ techniques or FN.

The study was approved by the Ethics Committee of the University of Paris-Saclay (File no: 2017/11). It is to be noted that a signed informed consent was not required from participants in this anonymous and voluntary questionnaire survey. Participants were informed that the return of the questionnaire signifies acceptance to participate.

### Questionnaire

Our anonymous questionnaire was distributed during a lecture after the provision of e-mail information, followed by another written explanation before data collection. In addition, the survey session was preceded by a verbal explanation, where students were told that they should not write their name on the questionnaire and that it was voluntary to return it. Those who wished to participate returned the questionnaire after approximately 45 min, with no researchers being able to see how they responded. The survey was conducted over three sessions in November and December 2017, followed by two extra sessions at the two sites in January 2018, for students who were absent at the first session, as identified from the roll call. These students were invited by e-mail to participate at this extra session, if they wished.

### Pilot study

A pilot study was conducted with eight students, who had passed their final exam but still had some clinical obligations. At least one of the authors was present when the students answered the questions, and the participants were encouraged to comment or ask questions relating to the questionnaire. This procedure resulted in some minor modifications to facilitate its use.

### Data collection

In addition to questions on *age, year of study, and campus site*, 10 questions relating to *chiropractic conservatism* were either selected from previous studies or created specifically for the present survey, as explained in detail elsewhere [14]. Answers deemed to indicate a conservative view were given 1 point and the others 0 points. The sum of all points for the ten questions (0–10 points) was converted into four chiropractic conservatism groups [1–4], with 4 indicating an extremely conservative view of chiropractic.

This score was developed and validated in a previous study originating from this survey. It showed that students with the higher scores were more unlikely to identify non-indications to chiropractic care than students with lower scores [14]. This finding was considered logical, as students, who believe that spinal manipulations (‘adjustments’) have an almost unlimited beneficial effect on bodily functions, would perceive very few limitations for such treatment, apart from contra-indications.

The survey included also a question about the desire to use ‘*prescriptive’ chiropractic techniques*: “When you have graduated would you like to use one or several specific chiropractic technique evaluation systems, which tell(s) you what the problem is. For example, SOT, Gonstead, Applied Kinesiology, or Functional Neurology”. Answering possibilities were: “Yes”, “Yes, probably”, “Don’t know”, “No, probably not”, “No”. This question was taken from a previous report on chiropractic students in Australia [11].

Included for this report were two sets of questions on *Functional Neurology* 1) to define the *level of awareness* of this concept and 2) to obtain an understanding of the students’ i) *interest in FN* and ii) *acceptance of* some basic concepts often encountered in FN.

The first set of questions on their *awareness* of Functional Neurology consisted of one question taken from another survey of chiropractic students [11] with eight answering possibilities ranging from “Never heard of Functional Neurology” to “Know Functional Neurology well”.

The second set of questions on Functional Neurology (*n* = 6), consisted of 3 questions on the *interest in* FN, taken from the same survey as the previous question [11] and 3 questions on *acceptance of* some concepts of FN created by the research team.

Questions on i) *interest* were:

- “Would you like to learn (more) how to use Functional Neurology?” (Yes/Unsure/No).
- “Do you think this technique should be taught in chiropractic programs?” (Yes mandatory/Yes as an elective only/Don’t know/Probably not/Definitely not).
- “Do you think that this technique holds great promise for chiropractic?” (Yes definitely/Yes probably/Don’t know/Probably not/Definitely not).

The two questions and one statement on ii) *accept* were:

- “In your opinion, can chiropractic spinal adjustments influence brain function?”, and …
- “Mrs X brings her son, Julien 7 yrs. to consult you. She explains that Julien is hyperactive and that she has read on the Internet that chiropractors can detect and treat groups of dysfunctional neurons in the brain, which could improve his hyperactivity. Would you accept to treat this boy in this way? (Definitely not/Probably not /Don’t know/Yes probably/Yes, definitely).
- “It is possible to examine the nervous system of young healthy people to detect groups of dysfunctional cerebral neurons.” (Strongly disagree/ Somewhat disagree/I don’t know/Somewhat agree/Strongly agree).

However, one of our newly created questions (“In your opinion, can chiropractic spinal adjustments influence brain function?”) was removed before analysis, because we realized that it had been too general. Any impulse to the body will be registered by the brain, and the question would therefore not be specific to the FN concepts. Thus, only five questions remained. The questions that were taken from the literature were translated into French and then back to English again by two bilingual persons.

In addition, there was one question about self-confidence. This was: “How do you think you will rate as a chiropractor compared to other chiropractors in your class?” Possible answers were “Below average”, “A bit below average”, “Average”, “A bit above average”, “Above average”, “I don’t know”. This question was used to compare the profile of the French chiropractic students to that of Australian students in a previous study, in which the same question had been used [11]. Translation and re-translation took place also here.

### Variables of interest and transformation of data

#### *Independent variable*

The 10 questions on chiropractic conservatism were dichotomized into ‘appropriate’ (0 point) and ‘inappropriate’ (1 point) answer, resulting in the ‘chiropractic conservatism score’. Based on the data distribution, this score was divided in four groups: group 1 (0–2 points), group 2 (3–5 points), group 3 (6–7 points), and group 4 (8–10 points), as previously defined [14]. The concept of ‘appropriate’ and ‘inappropriate’ was based on our opinion of modern, mainstream chiropractic.

#### *Dependent variables*

i) The use of ‘prescriptive’ chiropractic techniques

The answers on ‘prescriptive’ techniques were divided into two groups; The first group with “yes” and “yes, probably” indicated ‘interested’ and the second group with “don’t know”, “no, probably not” and “no” was interpreted as ‘uninterested’. The first group was given one point and the second group was given 0 points.

ii) Attitudes to Functional Neurology

The 5 questions used for the Functional Neurology score were transformed into dichotomous variables. ‘Negative answers’ were graded 0 and ‘positive answers’ were graded 1 (see Table 1). The total score per person (0–5) was thereafter transformed into a binary variable, with 0–1 considered to indicate that the student was mainly uninterested/disagreeing and with 2–5 considered to indicate interested/agreeing students.

**Table 1:** Description of questions on attitudes to (*interest in* and *accept of*) Functional Neurology and the scoring system in a survey of chiropractic students

***Acceptance of*** and ***interest in*** Functional Neurology	Answering possibilities				
‘Acceptance’ Mrs. X brings her son, Julien 7 yrs. to consult you. She explains that Julien is hyperactive and that she has read on the Internet that chiropractors can detect and treat groups of dysfunctional neurons in the brain, which could improve his hyperactivity. Would you accept to treat this boy in this way?	Definitely not	Probably not	Don’t know	Yes Probably	Yes, definitely
	Answers indicating disagreement with the concept			Answers indicating agreement with the concept	
	0 pt			1 pt	
‘Interest’ Would you like to learn (more) how to use Functional Neurology?	Yes	Unsure	No		
	Interested	Uninterested			
	1 pt	0 pt			
‘Interest’ Do you think this technique should be taught in chiropractic programmes?	Yes, mandatory	Yes, as an elective only	Don’t know	Probably not	Definitely not
	Interested		Uninterested		
	1 pt		0 pt		
‘Interest’ Do you think that this technique holds great promise for chiropractic?	Yes, definitely	Yes probably	Don’t know	Probably not	Definitely not
	Interested		Uninterested		
	1 pt		0 pt		
‘Acceptance’ It is possible to examine the nervous system of young healthy people to detect groups of dysfunctional cerebral neurons.	Strongly disagree	Somewhat disagree	I don’t know	Somewhat agree	Strongly agree
	Disagreement			Agreement	
	0 pt			1 pt	

#### *Additional variables*

*Sex* (male/female), *year of study* (3rd, 4th, 5th and 6th year), and *campus site* (Paris/Toulouse) were used for descriptive reasons, to compare responders to the profile of all students, and to control for potential modifying effects, whereas C*onfidence in self* with its six choices was treated only as a descriptive variable.

### Data analysis

Data were entered in EPIDATA 3.1. and double checked; one author (GG) reading the questionnaire with the pre-coded form and the other one (MD) entering the data, after which they switched roles. Analysis was done with STATA 15. Descriptive analyses were performed of each variable after which associations were tested between the independent variable (i.e. chiropractic conservatism score) and the dependent variables, (i.e. i) the desire to use ‘prescriptive’ chiropractic techniques and ii) the Functional Neurology score). This was done with logistic regression and reported as odds ratios (OR) with their 95% confidence intervals (CIs). The results were then adjusted for site (Paris or Toulouse), sex, and study grade (3rd – 6th yrs). Differences between groups were considered significant for *p* < 0.05 and when the 95% CIs did not overlap.

## Results

### Responders, response rate, description of participants

Of the 536 invited students, 359 (67%) participated in the survey. Sixty-seven percent were females and 55% were located at the Paris campus. There were approximately equal proportions of students from the different study years (years 3 to 6) (Table 2).

**Table 2:** Description of study participants in a survey of French chiropractic students

Variables	Numbers of responders (%)
**Sex (*N*** **= 359)**	
Females	241 (67)
Males	118 (33)
**Year of study program (*N*** **= 359)**	
3rd yr	102 (28)
4th yr	99 (28)
5th yr	72 (20)
6th yr	86 (24)
**Site of Campus (*N*** **= 359)**	
Paris	199 (55)
Toulouse	160 (45)
**Confidence in self (*N*** **= 353)**	
Below average	2 (0)
A bit below average	11 (3)
Average	155 (44)
A bit above average	90 (25)
Above average	22 (6)
Don’t know	73 (21)

Most of the students seem to have reasonable self-confidence, as they think that when graduated they would either be ‘average’ or more (75%) when compared to their colleagues (Table 2). The corresponding percentage was 79% in the previous Australian survey [11].

### Comparisons between responders and non-responders

As shown in Table 3, there were somewhat more responders in Toulouse than in Paris (80% vs. 59%), the 3rd year students were more likely to participate than the 5th year students (81% vs. 55%), and the participation rate was almost equal for the two sexes (males 60% vs. females 71%).

**Table 3:** Comparison of responders and non-responders in a survey of French chiropractic students (N = 359)

	Responders N (%)	Non responders N (%)	Total N (%)
**Sex**			
Male	118 (60)	79 (40)	197 (100)
Female	241 (71)	98 (29)	339 (100)
**Year of study**			
3rd year	102 (81)	24 (19)	126 (100)
4th year	99 (67)	48 (33)	147 (100)
5th year	72 (55)	60 (45)	132 (100)
6th year	86 (66)	45 (34)	131 (100)
**Site of campus**			
Paris	199 (59)	138 (41)	337 (100)
Toulouse	160 (80)	39 (20)	199 (100)

### Chiropractic conservatism score

Table 4 shows the spread of data for the ‘chiropractic conservatism score’, divided into four groups, from low (group 1) to very high (group 4). Over 70% can be described as ‘very conservative’ (groups 3 and 4) in relation to their approach to the health impact of the “subluxation” and the outstanding effect of the “chiropractic adjustment”. Only a minority of students are classified as only somewhat conservative (group 1). Compared to the 3rd year students, the 6th year students were about twice as often found in group 4 (31% vs 61%), with a statistically significant test of trend for increasing year of study (*p* = 0.002).

**Table 4:** Chiropractic conservatism score reported by year of study in a survey of French chiropractic students (*N* = 354)

	3rd year	4th year	5th year	6th year	Total
**Conservatism group 1**	6 (6%)	1 (1%)	4 (6%)	4 (5%)	***15 (4%)***
**Conservatism group 2**	24 (24%)	20 (20%)	16 (23%)	10 (12%)	***70 (20%)***
**Conservatism group 3**	40 (40%)	32 (33%)	27 (38%)	19 (23%)	***118 (33%)***
**Conservatism group 4**	31 (31%)	45 (46%)	24 (34%)	51 (61%)	***151 (43%)***
***Total***	***101 (101%)***	***98 (100%)***	***71 (101%)***	***84 (101%)***	***354***

### Outcome variables

#### *Interest in the use of ‘prescriptive’ techniques*

In all, 91% reported that they were interested in the use of ‘prescriptive’ techniques, with no significant difference between the study years (Table 5).

**Table 5:** Proportion of students who wish to use prescriptive techniques by year of study in a survey of French chiropractic students (*N* = 359)

	3rd year numerator/denominator (%)	4th year numerator/denominator (%)	5th year numerator/denominator (%)	6th year numerator/denominator (%)	Test for trend (*p*-value)	All numerator/denominator (%)
Q12: When you have graduated would you like to use one or several specific chiropractic technique evaluation systems which tell(s) you what the problem is. For example SOT, Gonstead, Applied Kinesiology, or Functional Neurology.	93/102 **(91)**	91/99 **(92)**	61/72 **(85)**	80/86 **(93)**	0.930	325/359 **(91)**

#### *Awareness of Functional Neurology*

Table 6 shows the answers to questions relating to the awareness of FN by year of study. Overall, only 10% had never heard of it, whereas about 50% indicated at least some awareness of it, selecting “heard about it”. There were some differences in the estimates for the awareness level between the study years, notably that no 6th year student had ‘never heard of it’ (column 5, row 2) vs. 20% in the 3rd year (column 2, row 2). The percentages in row 5 show that having read FN articles or been to seminars was reported by 13% in the 6th year vs. 1% in the 3rd year. A test for trend shows that there is a significant difference between year of study program (*p* = 0.000).

**Table 6:** Awareness of Functional Neurology reported by year of study in a survey of French chiropractic students (*N* = 358)

Q13: What is your level of awareness of Functional Neurology (FN)? Select the **single** best option from the following:	Year 3 N (%)	Year 4 N (%)	Year 5 N (%)	Year 6 N (%)	All N (%)
Never heard of it	20 (20)	12 (12)	3 (4)	0 (0)	**35 (10)**
Heard about it	61 (60)	50 (51)	40 (56)	33 (38)	**184 (51)**
Seen it used/ know a treated person	20 (20)	24 (24)	16 (22)	41 (48)	**101 (28)**
Know FN: read articles/ been at seminars	1 (1)	11 (11)	11 (15)	11 (13)	**34 (9)**
Know FN well	0 (0)	0 (0)	0 (0)	1 (1)	**1 (0)**
Others	0 (0)	1 (1)	2 (3)	0 (0)	**3 (1)**
**Total**	**102 (101)**	**98 (99)**	**72 (100)**	**86 (100)**	**358 (99)**

#### *Positive attitudes to Functional Neurology*

When enquiring about attitudes to concepts encountered within FN, as can be seen in Table 7, clearly there is a great interest in this approach, as almost all would like to learn more about it, and many think it should be included in the chiropractic undergraduate syllabus. However, only a minority would agree to treat a hyperactive child (Table 7, row 2, column 7), and the concept of “dysfunctional neurons” is accepted by even fewer students (Table 7, row 6, column 7). There was no obvious link to their level of knowledge in neurology, as the responses are independent of year of study.

**Table 7:** Distribution of positive attitudes to (i.e. *interest in* and *acceptance of*) Functional Neurology. The percentage of participants who answered ‘yes’ have been included in this table

*Acceptance of* and *interest in* Functional Neurology	3rd year numerator/denominator (%)	4th year numerator/denominator (%)	5th year numerator/denominator (%)	6th year numerator/enominator (%)	Test for trend Prob > z	All numerator/denominator (%)
‘Acceptance’ Q1: Mrs. X brings her son, Julien 7 yrs. to consult you. She explains that Julien is hyperactive and that she has read on the Internet that chiropractors can detect and treat groups of dysfunctional neurons in the brain, which could improve his hyperactivity. Would you accept to treat this boy in this way?	22/101 (22)	29/98 (30)	21/72 (29)	26/86 (30)	0.220	98/357 (27)
‘Interest’ Q2: Would you like to learn (more) how to use Functional Neurology?	94/101 (93)	91/99 (92)	68/71 (96)	83/86 (97)	0.208	336/357 (94)
‘Interest’ Q3: Do you think this technique should be taught in chiropractic programmes?	70/102 (69)	74/99 (75)	55/71 (77)	73/86 (85)	**0.009**	272/358 (76)
‘Interest’ Q4: Do you think that this technique holds great promise for chiropractic?	52/102 (51)	57/99 (58)	38/71 (54)	67/86 (78)	**0.001**	214/358 (60)
‘Acceptance’ Q5 It is possible to examine the nervous system of young healthy people to detect groups of dysfunctional cerebral neurones.	24/102 (24)	17/98 (17)	13/72 (18)	22/86 (26)	0.746	76/358 (21)

In sum, there were statistically significant trends in favour of a gradual increase in interest for FN with year of study (Table 7, column 6, rows 4 and 5) but not for acceptance of some concepts of treatment (Table 7, column 6, rows 2 and 6).

#### Functional Neurology score

When the numbers of responses indicating ‘agreement’ or ‘interest’ (=1 point each, as shown in Table 1) were added into a summary score (0–5) and then grouped into those scoring 0 to 1 vs. the others, it can be seen in Table 8 that almost all students (83%) have somewhat to very positive attitudes to FN. Thus only 17% have almost no or not positive attitudes to FN. In fact, only 14 individuals (4%) scored 0 points to these five questions.

**Table 8:** Description of the distribution of scores obtained in a survey of French chiropractic students indicating level of interest in and accept of Functional Neurology (N = 354)

	N (%)
**Functional Neurology score (*N*** **= 354)**	
0	14 (4)
1	45 (13)
2	67 (19)
3	131 (37)
4	68 (19)
5	29 (8)
**Functional Neurology score group (N = 354)**	
Group 1 (0–1)	59 (17)
Group 2 (2–5)	295 (83)

### Is adherence to chiropractic conservatism concepts associated with i) an interest in the use of ‘prescriptive’ chiropractic techniques and ii) a positive attitude to Functional Neurology?

#### *Interest in the use of ‘prescriptive’ chiropractic techniques*

As seen in Table 9, there is a positive association between the ‘conservatism score’ and the wish to use ‘prescriptive’ techniques. This association increases with increasing conservatism, i.e. there is a positive gradient. The unadjusted OR is 14, when comparing the lowest conservatism group with the highest, and the adjusted OR is 17, when controlling for sex, site and year of study. In other words, the most ‘chiropractically’ conservative students are 17 times more likely to want to use ‘prescriptive’ techniques than the least conservative students. The confidence intervals were well over 1 but rather wide.

**Table 9:** Bivariate and multivariate analyses testing the association between chiropractic conservatism and i) the use of prescriptive chiropractic techniques, and ii) positive attitudes to Functional Neurology in a survey of French chiropractic students (N = 354)

	Systematic chiropractic techniques (Yes/No) OR (CI 95%)		Functional Neurology score (0–1/2–5) OR (CI 95%)	
	Unadjusted	Adjusted	Unadjusted	Adjusted
**Conservative score**				
● **1 (reference)**	1	1	1	1
● **2**	6.78(1.93;23.75)	9.70 (2.41;39.05)	2.60(0.84;8.154)	2.60(0.76;8.89)
● **3**	12.03(3.47;41.68)	17.99(4.42;73.27)	7.07(2.25; 22,227)	7.13(2.09;24.26)
● **4**	13.81(4.09;46.66)	16.86(4.23;67.18)	12.04(3.76;38.598)	10.93(3.20;37.43)

#### *Positive attitudes to Functional Neurology*

Similarly, there is also a significant association between the ‘conservatism score’ and the ‘Functional Neurology score’. The unadjusted OR is 12 and the adjusted estimate is 11 (controlling for sex, site, and year of study). The estimates increased in a dose-response fashion but were significantly different from the index group (the lowest conservatism score group) only for the last two groups. Again, the confidence intervals were wide but well above 1 (Table 9).

## Discussion

The results of this survey confirmed that 3rd- 6th year chiropractic students in this institution are very interested in ‘prescriptive’ techniques. They also want to learn about FN, and they have a positive attitude to this technique, which show that FN is attractive for chiropractic students, even if it is not taught in their course. Furthermore, the more conservative they are in relation to older chiropractic concepts, the more interested in ‘prescriptive’ techniques they are and the more positive to FN. However, this interest in FN seems incongruous, as they in general do not really adhere to some of the concepts of FN.

### Comparisons to other studies and explanations

Chiropractic schools have different approaches. Some are more chiropractically ‘conservative’ than others. Not surprisingly, a study has shown that some chiropractic institutions appear to produce chiropractors with non-evidence-based beliefs and maybe even contrary to evidence (e.g. anti-vaccination) [17]. However, the present results are surprising, as the French chiropractic school, where this study took place, has an evidence-based approach and the curriculum does not include prescriptive techniques such as Applied Kinesiology and Functional Neurology.

It is probably common that chiropractic students, in general, look for advice in the treatment of patients through chiropractic ‘prescriptive’ techniques, whether simplistic or more complicated. Almost all participants were in favour of this, which is comparable to the findings of a previous survey of Australian chiropractic students from two university-based schools [11]. That this is strongly linked to chiropractic conservatism, however, has not been shown or even studied before.

It could be argued that the wording of our question made it almost impossible to say ‘no’ (“When you have graduated would you like to use one or several specific chiropractic technique evaluation systems, which tell(s) you what the problem is?) but we argue that at least students in the final years should have assimilated sufficient knowledge to be able to muster enough common sense to realize that this is not a realistic possibility. The fact that year of study was not protective against such beliefs and that the association pattern with the conservatism score resembled much that for the acceptance of Functional Neurology, make us conclude that most students probably understood what we meant, namely that there are diagnostic techniques that pretend to be able to much simplify some clinical decisions.

Interestingly, another strong link with chiropractic conservatism emerged also from this same survey, as reported elsewhere, namely that the more conservative students are less likely to identify reasonable limits of chiropractic treatment (non-indications) [14]. In other words, the more ‘chiropractically’ conservative the student is, the more likely to accept almost all patients for treatment and, as shown in the present report, to do so using stereotypical treatment approaches (‘prescriptive’ techniques).

These findings are intriguing. Some ‘prescriptive’ techniques are rather simple to learn and use whereas others are more complicated (i.e. FN); perhaps the common link is that they all systematize the treatment of many conditions whilst bypassing the depth of knowledge needed to fully understand such conditions. Clearly, holding an extreme chiropractic conservative view is likely to lead to a belief, for example, that spinal problems are the cause of many kinds of disease and that the removal of these spinal problems will have a positive effect on the whole body. In our opinion, these two approaches disregard generally accepted clinical and scientific concepts of today.

Interestingly, there was a difference between an *interest in* FN and an *acceptance of* some of its concepts, with fewer accepting (less than 30%) than showing an interest in FN (the majority). Further, the level of acceptance did not increase significantly with the year of study, whereas there was a significant increase, from about half to almost 80%, of those who were interested in FN in the last year of study. Perhaps an evidence-based curriculum helps students develop a critical sense in relation to unusual theories, even if they may be receptive to the attractive aspects of communication and images of techniques that promise a lot. It is, of course, also possible that students become increasingly keen to increase their clinical armamentarium towards the end of their studies and are merely interested to pick up various ‘tricks of the trade’ without wanting to adopt the entire package.

Since this information was obtained in a cross-sectional study, it is of course not possible to determine if students develop in a certain way, as they progress through their educational program, or if we merely observed a cohort effect. It is, however, curious, that the students in the higher classes, who should have accumulated most knowledge and clinical understanding, were most interested in both ‘prescriptive’ techniques and FN. It is disappointing that they, at this stage, have not better understood the complexities of the human body and its pathologies.

### Methodological considerations

This survey was anonymous and voluntary, and the response rate was reasonable (67%). Nevertheless, the non-responders could have been different from the responders, but as the attendance to courses is not obligatory and students often stay away, there were no obvious reasons to believe that the non-responders kept away from classes because of an opposition to this study. Although the proportion of participants was not equal between sites, study year and sexes, this is unlikely to bring a specific bias into this study. The number of unanswered questions was very low, for which reason we did not think that non-response or absence from the survey session was due to bias, such as a dislike of the survey questions.

We consider the validity of our survey instrument acceptable. The question about ‘prescriptive’ techniques was already used in a recent published survey of Australian students, with similar results to ours, indicating external validity [11]. This was also the case for the question on “confidence in self” [11].

Two of our questions on FN were not previously validated because we created them for the survey. However, they were direct questions and tested in a pilot study and found to be user-friendly [14]. In addition, the small number of missing answers indicates the same. FN appears to be a complex phenomenon, and other or more questions or examples could perhaps have brought other answers, but we had to keep the survey relatively brief to obtain a reasonable response rate and good compliance on the questions. It is also possible that the question on reliance on techniques could have yielded fewer positive answers had it been worded more critically, for example including words such as “purporting” or “pretending”. The strong association between conservatism and the interest in ‘prescriptive’ techniques indicates, however, that the interpretation of this question depended on the student and not so much the wording of the question.

The questions on conservatism used in this survey were previously reported and we considered the results to be logical [14], with higher conservatism scores being associated with an inability to accept that there are cases that are outside the scope of chiropractic practice.

Thus, in general our results appear logical but might have been somewhat different, if the cut-point for « yes » and « no » or classifications of data had been different. However, we did not perform sensitivity analyses to test this, because we considered our cut-points to be logical.

### Perspectives

The results of this survey are of concern for the School and groups within the profession. Repeat surveys at regular intervals are therefore recommended to establish if these profiles follow individual classes over time or if they depict a typical developmental pattern from the lower to the higher years. In addition, regular surveys could be used to monitor various pedagogic interventions, particularly interesting at this particular institution, as there are two campuses located in different parts of the country, making it possible to compare different interventions.

## Conclusion

In general, although ‘prescriptive’ techniques, including Functional Neurology, are not taught within the curriculum, the chiropractic students in a European chiropractic institution appeared to be interested, intrigued and maybe even attracted by the ideas behind these techniques. This interest increased gradually in students attending year 3 to those attending the final years of study. Further, and even more importantly, regardless year of study, the stronger their beliefs in the conservative subluxation concept, the more likely they were to opt for such ‘prescriptive’ techniques. Despite this considerable interest, students do not generally agree with some key concepts within Functional Neurology. It is therefore possible that the evidence-based curriculum of the School protects against illogical concepts among students but does not satisfy their curiosity regarding various chiropractic approaches, particularly towards graduation.

## Data Availability

The data used for the current study are available from the corresponding author on reasonable request.

## Abbreviations

FN: Functional Neurology
CI: Confidence Interval
SOT: Sacro-Occipital Technique
OR: Odd Ratio
